# Development and Validation of Online Textual Pediatrician-Parent Communication Instrument Based on the SEGUE Framework

**DOI:** 10.1155/2019/8638174

**Published:** 2019-04-28

**Authors:** Yuqi Xiong, Dan Wang, Haihong Chen, Xuemei Wang, Xinping Zhang

**Affiliations:** School of Medicine and Health Management, Tongji Medical College, Huazhong University of Science and Technology, Wuhan 430030, China

## Abstract

The prevalence and feature of online textual pediatrician-parent communication (OPPC) have been recognized, but evidence on OPPC assessment remains insufficient. This study aimed to develop and validate an OPPC instrument to provide measurement and quality characteristics for quality assessment and management. 490 OPPC exchanges of 61 tertiary hospitals from 9 Chinese provinces were obtained from the Spring Rain Doctor website. The SEGUE framework, OPPC feature, and a pilot study were considered to establish the instrument. An empirical study was conducted to validate it and the incidence of OPPC items was also analyzed. As a result, a four-dimensional, 15-item OPPC instrument was developed. The empirical results are as follows. Cronbach's *α* values of dimensions were 0.80, 0.62, 0.64, and 0.60; the mean interrater reliability was 0.93; the correlation coefficients between items and their corresponding dimensions' scores ranged from 0.51 to 0.89 (P<0.001). The goodness-of-fit indices were acceptable. The overall incidence rate of parent-dominated/cooperative items (46.9%) was higher than that of pediatrician-dominated items (39.6%). Thus, the instrument is acceptable and OPPC quality is characterized by more parent-dominated and cooperative behaviors.

## 1. Introduction

With the ease of internet access and growing patient consumerism, the channels that people use to seek advice on medical problems have been moving from traditional face-to-face communication to online communication, making online health communication management a new but difficult issue in the field of health policy and management worldwide [1–4]. Most online physician-patient communications occur when patients have not consulted a physician face-to-face or are uncertain or dissatisfied with the diagnosis or treatment after consulting a physician [5, 6]. People may also choose online communication when they perceive their problems as noncritical [7, 8]. Although online communications via telephone, e-mail, and even remote video conferencing become more prevalent in many countries or regions, textual communication is still a common form of online communication in some developing countries such as China where it lacks the flexible or efficient use of various communication means [9, 10]. An asynchronous, online textual communication has several advantages: the first is convenience; that is, people can easily choose their communication partners and use the service whenever and wherever they prefer, as long as internet connection is available [11]; the second is enhanced controllability of self-presentation, and partners can carefully present themselves well [11–13]; the third is source and audiovisual anonymity, thereby resulting in the usage of more verbal cues, such as direct questions and answers [11, 13]. As a result, people feel more comfortable and may participate more actively for accurate diagnosis and effective treatment in online textual communication than in face-to-face communication [13].

For pediatrician-parent communication, more parent's and cooperative communication behaviors occur than pediatrician's behaviors, thereby reflecting pediatricians' respect for parents' expertise in caring for their children and the importance of parent participation in communication [14]. Parent-dominated communication behavior is the behavior led by the parent [15]. In parent-dominated communication behaviors, parents actively start the topic in the communication process (e.g., a parent takes the initiative to inform the pediatrician about disease progression before the physician asks such questions) [15]. Cooperative communication behavior is that completed by both participants (e.g., parent and pediatrician discuss how health problems affect the child's life) [15, 16]. Communications characterized by parent-dominated and cooperative behaviors usually contribute to good communication quality [17]. However, reports on measurement and quality characteristics of online pediatrician-parent communication (OPPC) with considering the OPPC feature are insufficient. In this study, when it says OPPC, it refers to online textual pediatrician-parent communication for simplicity.

Attempts have been made worldwide to assess the process of face-to-face physician-patient communication by using several instruments [18, 19]. Among the existing observer-coding instruments (e.g., RIAS, VR-MICS, PPCP, CSBL, and SEGUE), the SEGUE framework is a research-based checklist with good methodological quality evaluated by the Consensus-Based Standards for Selection of Health Measurement Instruments guidelines [19]. The SEGUE framework has been used widely in a variety of contexts [18]. Assis-Hassid assessed physician's communication skills in a computer setting by using the SEGUE framework (interrater reliability: Cohen's k=0.515) [20]. Then, this author's subsequent study provided an initial validation process of an e-SEGUE in assessing physician-patient communication with good content validity [21]. Braverman characterized clerkship students' perspectives on communication challenges in the outpatient setting by using the SEGUE framework (interrater reliability: Cohen's k=0.85) [22]. Sattler compared actual patient and standardized patient assessments of medical students' communication skills by using a modified SEGUE framework for its readability without verifying reliability and validity [23]. In China, researchers have also used SEGUE or modified SEGUE to assess face-to-face communication skills (Cronbach alpha: 0.83–0.88; Kaiser–Meyer–Olkin: 0.74–0.92) [24, 25]. Interestingly, research on instruments to assess online physician (pediatrician)–patient (parent) communication is limited. Although SEGUE was originally developed to assess face-to-face communication, it contains important dimensions of online communication and may be modified to assess online communication due to its good reliability, validity, and usability [16].

Although several studies explored quality assessment of face-to-face physician-patient communication process, few have explored quality assessment of OPPC, despite its importance and complexity [10, 26]. This study aimed to fill this information gap by developing and validating an OPPC instrument based on the SEGUE framework, taking the OPPC feature into consideration. The established instrument was expected to be used to measure the OPPC process and provide quality characteristics of OPPC process for quality assessment, improvement, and management.

## 2. Methods 

### 2.1. Study Setting and Sampling

Data were collected from a retrospective review of the online platform “Spring Rain Doctor” for physician-patient communication. The Spring Rain Doctor website (https://www.chunyuyisheng.com/) is one of the largest websites serving for health consultations. By August 2017, this website has covered 17 clinical departments, attracted over 50 million physicians, and served over 200 million people [27]. The website provides two fundamental services: (1) health information (medical knowledge, news, and expert opinions) search and (2) health communication between physicians and patients (via text, picture, or voice messages) [27]. In this study, an OPPC exchange means such a health communication process. The data of the OPPC exchanges in this study is reported on the website in public while the content is anonymous for health information sharing.

Nine provinces (i.e., eastern China: Zhejiang, Beijing, and Liaoning; central China: Hubei, Henan, and Anhui; western China: Sichuan, Shanxi, and Guizhou) were selected by considering their gross domestic product, geographical position, and data availability. Communications concerning adult treatments were excluded from the study. From the latest OPPC exchange ending in March 20, 2018, we selected every 5th exchange and five exchanges for each pediatrician. Finally, the full sample included 490 communication exchanges between parents and 98 pediatricians from 61 tertiary hospitals. These pediatricians included were mostly female (65.3%). The titles of the 98 pediatricians included physician (53.1%), physician-in-charge (32.6%), associate chief physician (10.2%), and chief physician (4.1%).

### 2.2. Instrument Design: The SEGUE Framework

The SEGUE framework focuses on a complete communication process and divides a communication into five dimensions, that is, “Set the stage,” “Elicit information,” “Give information,” “Understand the patient's perspective,” and “End the encounter”, comprising 25 items. “Set the stage” refers to the stage of patient's opening statement about his/her concerns; “Elicit information” refers to the stage of eliciting and exploring patient's full set of problems, such as factors and effects of the problems; “Give information” refers to the stage of physician providing the information about the patient's health condition; “Understand the patient's perspective” refers to the stage of physicians' understanding of the patient's efforts and concerns and establishing relationship with their patients which may run through the whole communication process. At the stage of “End the encounter,” the physician asks if the patient would like to discuss anything else and reviews the next steps with the patient [16]. The five dimensions of SEGUE were initially used as OPPC dimensions because of their good reliability, validity, and usability [19]. Then these dimensions were confirmed or deleted by a pilot study which will be described later.

### 2.3. Item Generation

The OPPC instrument was designed as a reflective measurement according to Peterson's study [28] and described manifestations of the communication quality of each OPPC dimension. All OPPC items were generated as reflections of their corresponding dimensions. On the basis of original items of SEGUE and relevant literature of OPPC, the initial OPPC instrument comprising 29 items was established. All the five dimensions of SEGUE were temporarily retained at this stage.

The items were clear coded which contributed to good interrater reliability [16]. Most items were measured by whether the described behavior occurred in practice following the coding methods in SEGUE, as “Yes” indicating the described behavior did occur and “No” otherwise. Items of “Frequencies of social talks (pediatrician)” and “Frequencies of social talks (parent)” were measured by their corresponding frequency of words in communication practice, such as greetings, thanks, and emoji [29].

### 2.4. Pilot Study

To correct any possible problem of the OPPC items, a pilot study was conducted by using a sample of 40 OPPC exchanges. Items that meet both the following criteria were deleted [30]: (1) the deletion of the item resulted in increased Cronbach's *α* value of the whole instrument and (2) corrected item−total correlation < 0.3. The OPPC dimensions were confirmed or deleted based on their retained items.

### 2.5. Empirical Study: Content and Construct Validity and Reliability

An empirical study with 450 OPPC exchanges was conducted to validate the instrument. As the scores of items were mostly binary, therefore Spearman rank correlation analysis among the scores of items and their corresponding dimensions was also carried out to assess the content validity.

The exploratory factor analysis (EFA) aimed to explore the number of underlying dimensions that may be present in the data on the basis of the Kaiser principles [31] and Scree test [32]. The confirmatory factor analysis (CFA) aimed to examine the factor structure of the OPPC. As most OPPC items were binary variables, therefore the EFA and CFA were conducted with MPLUS, using weighted least squares with adjusted mean and variance, which is a robust method for binary variables [33, 34].

Reliability was determined by calculating Cronbach's *α* value and the interrater reliability. To test interrater reliability, we double-coded 30 randomly selected OPPC exchanges. As proposed by Ong et al. [35], the interrater reliability was calculated by means of Spearman rank correlation coefficients. A third reviewer helped make a decision in case of interrater disagreement to control assessment quality.

The goodness-of-fit indices, including Tucker–Lewis index (TLI: >0.90 acceptable), comparative fit index (CFI: >0.90 acceptable), and the root mean square error of approximation (RMSEA: <0.08 acceptable), were examined for the fitness evaluation of CFA [36, 37].

All statistical analyses were performed with MPLUS 7.0 and SPSS 13.0.

### 2.6. OPPC Quality Characteristics

OPPC quality characteristics were also measured by the incidence of OPPC items in the empirical study [38, 39]. We predefined the OPPC items as parent-dominated, pediatrician-dominated, or cooperative items. Items categorization was based on the initiator of the communication behavior of the corresponding item described. For example, the initiator of item “Parent's explanation of initiation of symptoms or disease” is the parent, so, this item was categorized as parent-dominated. The categorization of the other items was presented in Table 1.

By using the 490 samples, we then calculated, (1) for each binary coded item, the incidence rate of each item which was calculated by the number of “YES” of the item/490, (2) for each frequency coded item, the mean value of total frequencies which was calculated by the sum of frequencies/490, and (3) the overall incidence rates of parent-dominated/ cooperative items and pediatrician-dominated items, respectively, which was calculated by the sum of the numbers of “YES” of such items/(the number of such items *∗* 490).

## 3. Results 

### 3.1. Instrument Design, Item Generation, and Pilot Study

According to the item deletion principles mentioned in Section 2.4, 4 dimensions comprising 15 items were finally included and all items of “End the encounter” were deleted (Table 1, the Appendix section). “Elicit information” items covered the highest proportion of the OPPC items (40%). Cronbach's *α* values for each dimension in the pilot study were improved from 0.56, 0.62, 0.31, 0.66, and 0.07 to 0.76, 0.75, 0.64, and 0.70, respectively.

### 3.2. Empirical Study: Content and Construct Validity and Reliability

The correlation coefficients of items and their corresponding dimensions' scores ranged from 0.51 to 0.89 (P < 0.001). Items 1, 2, 3, 10, 11, 14, and 15 showed high correlation with their corresponding dimensions' scores (r > 0.80) (Table 2). The Kaiser–Meyer–Olkin value for EFA was 0.60, and the Bartlett test showed statistical significance (P < 0.001). The EFA results supported the construct validity of the modified OPPC instrument which were presented in Table 3. Although items 7 and 12 have relatively low loadings on their corresponding dimensions as well as relatively low correlation with their corresponding dimensions, the two items were kept for the following reasons. First, discussing the physical/physiological factors of the illness is necessary for the pediatrician to learn about the child's illness [40–42]. Second, the child's health status is one of the most important points parents care about [14]. Third, at least three items should be included in a dimension [43]. Overall, the CFA results showed acceptable fit to the data with three pairs of error variance captured under the dimension of “Elicit information” (TLI = 0.92, CFI =0.93, RMSEA = 0.11).

Cronbach's *α* values of dimensions in the empirical study were as follows: (1) “Set the stage,” 0.80, (2) “Elicit information,” 0.62, (3) “Give information,” 0.64, and (4) “Understand the patient's perspective,” 0.60. The interrater reliability was good, with the correlation coefficients ranging from 0.85 to 1.00 (r_s_ = 0.93 in average, P < 0.01) for each item.

### 3.3. OPPC Quality Characteristics

The incidence of OPPC items in the 490 OPPC exchanges was shown in Table 1. Excluding items 14 and 15 which were measured by frequency, the overall incidence rate of parent-dominated and cooperative items (46.9%) was higher than that of pediatrician-dominated items (39.6%). The highest incidence rate of item in the 490 OPPC exchanges (71%) occurred in item 13, a parent-dominated item, and the lowest incidence rate (21%) occurred in item 4, a pediatrician-dominated item.

## 4. Discussion

The study established and validated a concise and convenient instrument to assess OPPC which the previous studies had seldom shed light on.

*OPPC Instrument Possessed Good Content and Construct Validity with Distinct OPPC Feature*. The dimension “End the encounter” of the original SEGUE framework was deleted in the OPPC instrument possibly because the online format rarely resulted in parents' raising new concerns or introducing off-topic talk. Thus, the pediatrician did not need to take steps to cut communication short [44].

It is interesting to find that instrumental behaviors or medical talks played a more important role than affective behaviors in OPPC as “Elicit information” items covered a large proportion of the instrument (40%). For this, the absence of physical examination in OPPC may provide a good explanation [45, 46]. As physical examination is the primary diagnostic means during face-to-face communications, especially in pediatrics, therefore, questions about the child's condition should be essential replacement of physical examination in OPPC [45, 46]. Otherwise, the whole OPPC would have been ineffective and even dangerous. This result can also be explained by the fact that computer-mediated communication was a cold and impersonal medium, in which emotions were more difficult to express timely and abundantly than traditional face-to-face communication, thereby reflecting the considerable importance of instrumental communication behaviors in an online format [47, 48]. This study suggested that pediatricians took more time in eliciting information on the causes of disease, especially physical and physiological factors, and the child's lifestyle. This finding agreed with the results of earlier studies, thereby indicating that physical and physiological factors determined the diagnosis and choice of treatment, and lifestyle was an important factor affecting the child's physical development [40–42]. What is more, through discussions, the pediatrician can elicit important and sufficient information through parent participation for accurate diagnosis and effective treatment [17]. As expected, this result indicated the necessity of information eliciting in OPPC.

Results from EFA and CFA analyses indicated good construct validity of the four-dimensional OPCC instrument. The OPPC dimensions reflected important aspects of a complete OPPC. Specially, the dimensions of “Give information” and “Understand the patient's perspective” were both parent-centered dimensions [49]. Communication patterns characterized as parent-centered were generally associated with better parent satisfaction, compliance, and medical outcomes as previous studies proposed [17]. Although “Give information” and “Understand the patient's perspective” were important aspects in most physician-patient/parent communication [50], the OPPC items showed what types of information should be given with parent participation and what behaviors were most important and encouraged to improve understanding in OPPC.

*OPPC Instrument Possessed Acceptable but Unexceptional Reliability*. Cronbach's reliability of instrument was acceptable but unexceptional possibly because the OPPC instrument lacked items about eyes and facial expressions which also reflected communication quality because they were unavailable in online practice [51, 52]. Another reason may be that most items were coded as binary variables which may increase random error [53]. However, the excellent interrater reliability indicated the usability of this observer-used instrument.

*A High-Quality OPPC Was Characterized by More Parent-Dominated and Cooperative Behaviors*. Unlike some studies which characterized communication quality as patient-dominated, physician-dominated, or cooperative by calculating amount of words of each partner in the communication process [54, 55], this study characterized OPPC quality by analyzing the incidence of item in the empirical study according to the principles proposed by October and Gregory [38, 56] to ensure the applicability and generalizability of this instrument in practice. The high incidence of parent-dominated and cooperative communication behaviors indicated that not only pediatricians' behaviors but the behaviors of both the participants deserve more attention for OPPC quality improvement and management. Results from the incidence of OPPC items further demonstrated the OPPC feature as previous studies proposed [14, 17].

## 5. Practical Implication

The study has several practical implications. Both the managers of online health communication platforms and policy makers can use this instrument to assess, regulate, and improve the OPPC practice. Considering that the instrument was developed and validated by multiple methods (pilot study, validity test, and reliability test), it may also be used for communication skills training of pediatric medical students in OPPC. In a high-quality OPPC process, parents should dominate the communication and focus their talks on their children's symptoms or initiation of the disease, symptoms or disease duration, and disease progression at the beginning of communication. At “Elicit information” stage, a cooperative way is encouraged. Pediatricians should focus on asking about the causes and effects of children's illness, especially for physical/physiological factors and lifestyle, and discuss such information with the parent. Parent participation is also encouraged at the stage of “Give information.” Information about physical examination results and children's health condition should be provided with priority. Specially, the pediatrician should not be the only one to educate the parent about physical examination results; parents are also encouraged to volunteer such information which was examined in hospital. Social talks of both the pediatrician and the parent should be used, such as greetings and thanks, to build a good pediatrician-parent relationship for good communication. Parents are suggested to acknowledge pediatricians' effort.

Although a different set of communication skills may be required with the coming of the new technology such as video-based communication, the communication content such as the causes and effects of the child's illness, and especially physical/physiological factors and lifestyle, should be similar to that of online textual communication. This will be the focus of future robustness studies of the OPPC instrument.

## 6. Limitations and Future Studies

This study has some limitations. First, the study was conducted in China where tensions between the physician and the patient/parent have risen to “irreconcilable edge” [57]. Whether the OPPC instrument is applicable for other countries with good physician-patient communication remains to be examined. Second, we cannot consider the relationship between parents and pediatricians in this study because of the limitations of the online health communication platform. This drawback may influence the communication content.

Future studies may focus on applying the OPPC instrument in assessing and comparing the online communication quality of various pediatric diseases or online health communication platforms to provide evidence for management such as transparency regulation. Studies on exploring the direct and indirect associations among the OPPC dimensions and standardization of OPPC process for effective communication are required. Then the results may be applied to quality evaluation, management, and decision making of online communication between physicians and parents or caregivers of patients who are unable to describe their disease clearly (e.g., Alzheimer's disease or psychiatric patients).

## Figures and Tables

**Table 1:** Dimensions and items of the OPPC instrument and the incidence of OPPC items.

Dimensions	OPPC items	Category ^a^	Incidence rate ^b^ (%) or mean ^c^
Set the stage	1. *Parent*'s explanation of initiation of symptoms or disease	①	36^b^
	2. *Parent*'s explanation of symptoms or disease duration	①	36^b^
	3. *Parent*'s explanation of disease progression	①	39^b^

Elicit information	4. *Pediatrician* asks parent how health problems affect the child's life	②	21^b^
	5. *Discuss* how health problems affect the child's life	③	29^b^
	6. *Pediatrician* asks about physical/physiological factors	②	31^b^
	7. *Discuss* physical/physiological factors	③	41^b^
	8. *Pediatrician* asks about how the child's lifestyle affects health problems	②	22^b^
	9. *Discuss* the child's lifestyle relevant to health problems with parent	③	32^b^

Give information	10. *Pediatrician* educates parents about the child's health according to physical examination results	②	57^b^
	11. *Parent* gives information about physical examination results	①	56^b^
	12. *Pediatrician* gives information about the child's health status	②	68^b^

Understand the patient's perspective	13. *Parent* acknowledges pediatrician's effort	①	71^b^
	14. Frequencies of social talks (*pediatrician*)	②	2.18 ^c^
	15. Frequencies of social talks (*parent*)	①	1.91 ^c^

The overall incidence rate of parent-dominated and cooperative items			46.9 ^d^
The overall incidence rate of pediatrician-dominated items			39.6 ^e^

^a^  ① parent-dominated item, ② pediatrician-dominated item, and ③ cooperative item.

^b^ Calculated by the number of “YES” of the item/490.

^c^ Calculated by the sum of the frequencies of social talks/490.

^d^ Calculated by the sum of the numbers of “YES” of parent-dominated and cooperative items/ (the number of parent-dominated and cooperative items *∗* 490).

^e^ Calculated by the sum of the numbers of “YES” of pediatrician-dominated items/ (the number of pediatrician-dominated items *∗* 490).

**Table 2:** The correlation of items and their corresponding dimensions' scores in the OPPC instrument.

No. of item	r	p
1	0.85	<0.001
2	0.81	<0.001
3	0.83	<0.001
4	0.51	<0.001
5	0.56	<0.001
6	0.61	<0.001
7	0.55	<0.001
8	0.56	<0.001
9	0.56	<0.001
10	0.89	<0.001
11	0.84	<0.001
12	0.54	<0.001
13	0.67	<0.001
14	0.85	<0.001
15	0.89	<0.001

**Table 3:** Factors and loadings of the four-dimensional OPPC instrument.

No. of item	Factor 1	Factor 2	Factor 3	Factor 4
Set the stage				
1	*0.843*	-0.097	0.126	0.027
2	*0.791*	0.007	-0.022	0.013
3	*0.791*	-0.072	0.156	0.004
Elicit information				
4	0.271	*0.611*	-0.238	0.132
5	0.294	*0.620*	-0.175	0.105
6	-0.140	*0.552*	-0.017	0.037
7	-0.069	*0.383*	-0.049	0.061
8	-0.135	*0.704*	0.253	-0.148
9	-0.106	*0.623*	0.303	-0.085
Give information				
10	-0.047	-0.077	*0.874*	0.072
11	0.060	-0.045	*0.879*	0.029
12	0.116	0.081	*0.323*	0.151
Understand the patient's perspective				
13	0.043	0.042	0.010	*0.711*
14	-0.079	0.114	0.068	*0.778*
15	0.078	-0.069	0.172	*0.809*

**Table 4:** The four-dimensional OPPC instrument.

No.	Item	Measure
	*Set the stage*	
1	Parent's explanation of initiation of symptoms or disease	Yes/ No
2	Parent's explanation of symptoms or disease duration	Yes/ No
3	Parent's explanation of disease progression	Yes/ No
	*Elicit information*	
4	Pediatrician asks parent how health problems affect the child's life	Yes/ No
5	Discuss how health problems affect the child's life	Yes/ No
6	Pediatrician asks about physical/physiological factors	Yes/ No
7	Discuss physical/physiological factors	Yes/ No
8	Pediatrician asks about how the child's lifestyle affects health problems	Yes/ No
9	Discuss the child's lifestyle relevant to health problems with parent	Yes/ No
	*Give information*	
10	Pediatrician educates parents about the child's health according to physical examination results	Yes/ No
11	Pediatrician gives information about the child's health status	Yes/ No
12	Parent gives information about physical examination results	Yes/ No
	*Understand the patient's perspective*	
13	Parent acknowledges pediatrician's effort	Yes/ No
14	Frequencies of social talks (pediatrician)	Number
15	Frequencies of social talks (parent)	Number

## Data Availability

The data used to support the findings of this study are available from the corresponding author upon request.

## Appendix

See Table 4.
